# Commentary: Use of Cannabinoids to Treat Acute Respiratory Distress Syndrome and Cytokine Storm Associated With Coronavirus Disease-2019

**DOI:** 10.3389/fphar.2021.631646

**Published:** 2021-04-12

**Authors:** Maurizio Bifulco, Donatella Fiore, Chiara Piscopo, Patrizia Gazzerro, Maria Chiara Proto

**Affiliations:** ^1^ Department of Molecular Medicine and Medical Biotechnologies, University of Naples “Federico II”, Naples, Italy; ^2^ Department of Pharmacy, University of Salerno, Fisciano, Italy

**Keywords:** cannabinoids, cannabidiol, SARS–CoV–2, COVID–19, pneumonia, ARDS

*by Nagarkatti, P., Miranda, K., and Nagarkatti, M. (2020) Front. Pharmacol. 11:589438. doi:* *10.3389/fphar.2020.589438*

We read with great interest the recent opinion by Nagarkatti et al. (2020), highlighting a potential role of cannabinoids in the treatment of acute respiratory distress syndrome (ARDS) associated with COVID-19. In particular, based on their previous studies evaluating the effect of THC in ARDS animal models, they focused the attention on the cannabinoid receptors targeting to control the hyperimmune response in severe COVID-19. Although cannabinoids and CBD in particular show an interesting potential, important issues concerning this therapeutics must be considered.

In recent months, the pressing need for effective treatments to counteract the spread of the COVID-19 pandemic dictated the development of new therapeutic approaches to handle or possibly prevent the complications of SARS-CoV-2 infections as a worldwide priority. Clinical profiles of COVID-19 patients range from asymptomatic infection to severe pneumonia with multisystem failure, the leading cause of mortality. In patients with severe disease, the occurrence of cytokine storm and a state of hyperinflammation led to acute respiratory distress syndrome (ARDS) (Lotfi and Rezaei, 2020). As Nagarkatti and colleagues (2020) highlighted, the potential use of cannabinoids in COVID-19 has been suggested for their immunomodulatory and anti-inflammatory properties, but not for the direct antiviral activity. Several authors focused the attention on the nonpsychoactive CBD as adjuvant in SARS-CoV-2 therapy. Recently, for the first time, it has been reported that CBD is able to reduce pro-inflammatory cytokine levels ameliorating symptoms of ARDS induced in a murine model (Khodadadi et al., 2020). Moreover, CBD seems to down-regulate the expression of ACE2 and TMPRSS2, two receptors exploited by SARS-CoV-2 to enter the cells (Wang et al., 2020). However, further studies to support CBD-mediated regulation of ACE2 and TMPRSS2 are needed.

Despite the encouraging potential of CBD, in our opinion, the first issue to consider is that, to date, there are no clinical data about the optimal anti-inflammatory dose and regimen of CBD in patients. Our knowledge about CBD use in patients comes mainly from few clinical studies evaluating the safety and efficacy of CBD as oral solution in the treatment of serious seizure disorders. The results from these studies highlighted that in comparison with other drugs employed for the treatment of seizure disorders, CBD has an overall safe profile, generally showing mild/moderate adverse effects (AEs). However, although with a low incidence, serious CBD AEs were registered (Brown and Winterstein, 2019; Huestis et al., 2019; Chesney et al., 2020; Dos Santos et al., 2020), some of which deserve particular caution in COVID-19 patients. The CBD-mediated impairment of immune response increases the risk of pneumonia or viral infection. Thus, particular attention must be paid for patients receiving immunosuppressive therapy, as some SARS-CoV-2 patients (Brown and Winterstein, 2019). Most importantly, it was observed that increased transaminases levels (ALT and AST) and hepatic injuries occur in CBD-treated patients who are chronically exposed to antiepileptic drugs, probably due to the multiple drug–drug interactions of CBD (Brown and Winterstein, 2019; Dos Santos et al., 2020).

CBD influences the principal enzymes (e.g., CYP450-3A4, -2C19, and UGTs) responsible for biotransformation of a wide range of drugs, thus potentially having impact on their pharmacokinetics and pharmacodynamics (Brown and Winterstein, 2019). The hypothetic drug–drug interactions of THC and CBD with the drugs currently used in therapeutic protocols for COVID-19, mainly antiviral and immunosuppressive drugs, have been analyzed (Land et al., 2020). However, the clinical profiles of frail patients infected by COVID-19 must be considered. Nowadays, the majority of patients included in CBD clinical trials are children or young adults. ARDS arises in severe COVID-19, and it is now clear that advanced age and several comorbidities including diabetes, hypertension, obesity and cardiovascular diseases are associated with disease severity, and predispose to a worse prognosis (Lotfi and Rezaei, 2020). This implies that with high probability, the COVID-19 patients with ARDS are under chronic therapies to treat their comorbidities. In this frame, we need to take into account the potential interaction of CBD with therapeutics like antiplatelet, antiarrhythmic, antihypertensive, or lipid-lowering drugs like statins, some of which are metabolized by CYP450 and/or UGTs (Brown and Winterstein, 2019), to avoid the worsening of liver and kidney injuries in COVID-19 patients (Lotfi and Rezaei, 2020).

Last but not least, it is reported that to exert their action, some cannabinoids require membrane lipid rafts integrity (Sarnataro et al., 2006), where cannabinoid receptors are localized. To produce its proapoptotic effect in murine primary microglial cells, CBD induces a lipid rafts coalescence, an event specifically reverted by the cholesterol-depleting agent methyl-β-cyclodextrin (Wu et al., 2012), suggesting the key role of lipid rafts in CBD signaling. Even if the anti-inflammatory action of CBD seems to be cannabinoid receptor independent and considering that ACE2 receptor reside into lipid rafts, further investigations are needed to evaluate the potential impact of CBD on SARS-CoV-2–host cell interaction.

The current global emergency dictates the identification of therapeutics suitable to counteract the COVID-19 infection. CBD shows an interesting potential, but it is clear that further studies are required to corroborate this hypothesis, encompassing a clinical evaluation of risks and benefits of CBD use in SARS-CoV-2 patients.
